# Kinematic running resistance of an urban rail vehicle undercarriage: a study of the impact of wheel design

**DOI:** 10.1038/s41598-023-37640-w

**Published:** 2023-07-05

**Authors:** Stanislav Semenov, Evgeny Mikhailov, Maxim Kovtanets, Oksana Sergienko, Ján Dižo, Miroslav Blatnický, Juraj Gerlici, Mariusz Kostrzewski

**Affiliations:** 1 Faculty of Transport and Building, Department of Logistics and Traffic Safety, Volodymyr Dahl East Ukrainian National University, Ioanna Pavla II Str., 17, Kyiv, 01042 Ukraine; 2 Department of Transport and Handling Machines, Faculty of Mechanical Engineering, University of Žilina, Univerzitná 8215/1, Žilina, 01026 Slovak Republic; 3 Division for Construction and Operation of Transport Means, Faculty of Transport, Warsaw University of Technology, Koszykowa 75, 00-662, Warsaw, Poland

**Keywords:** Mechanical engineering, Applied mathematics

## Abstract

Urban railway vehicles are important means of transport in towns and cities due to their high capacity, power source, and low running resistance, which make them efficient for operation. Although these properties are considered advantages, there is still room for improvement in their operational efficiency. The main objective of this article is to investigate the impact of railway wheel design on the level of kinematic running resistance, which is expressed as the amount of mechanical energy losses during the interaction of wheels with rails. This research focuses on simulation computations of two variants of wheel design schemes: the traditional design scheme (TKS) and a perspective design scheme (PKS) characterized by a rotating flange independently of the wheel tread surface. Two undercarriage multibody models have been created, one with TKS and one with PKS, and simulation computations have been performed for running speeds of 10 km/h, 20 km/h, 30 km/h, and 40 km/h on track models in curves with radii of 20, 50, 100, 150, 200, and 250 m. The evaluated indicators affecting the level of mechanical energy losses were creep forces, slip velocities, and average power. The most important findings of this study are that the PKS design scheme resulted in lower values of all assessed parameters.

## Introduction

An urban rail vehicle, commonly known as a tram, is one of the important types of urban railway transport vehicles, that is vital to the success of transportation systems in large cities. In this regard, tram traffic is actively developing all over the world^1–3^. The analysis of scientific and technical information shows that various technical solutions and innovations in the field of railway transport are usually implemented first in urban rail vehicles and later in other types of rail vehicles^4,5^. This trend also applies to the current development direction of railway transport, such as the low floor concept, which significantly impacts the design of undercarriages, traction drive-trains, brake systems^6,7^, and other components of railway vehicles^8,9^. It is known that the wheelsets of railway vehicles must support the vertical loads and the guiding forces while transmitting traction and braking forces in the wheel/rail contact^10–13^.

However, one disadvantage of standard wheelsets, where wheels are rigidly mounted to an axle, is their inability to facilitate low-floor trams. Recently, a low floor is increasingly important for the convenient use of urban rail vehicles in cities by individuals with disabilities, the elderly, and those with baby carriages^14,15^. In the case of a standard railway vehicle, the floor of a car undercarriage cannot be lowered due to the design of the standard wheelset, which comprises two railway wheels rigidly mounted on an axle. To overcome this limitation, a wheelset design inspired by road vehicles with independently suspended wheels has been proposed. This technical solution is known as the wheelset with independently rotating wheels (IRWs).

This research presents an alternative approach to reduce running resistance, which involves a technical solution for railway wheel design. The solution combines the independent movement of two key elements of the railway wheel: the tread surface and the flange, resulting in a perspective construction scheme abbreviated as PKS. The main focus of the research was to analyze the potential for reducing the kinematic running resistance of urban railway undercarriages by implementing PKS wheels.

The design of a wheelset with independently rotating wheels completely changes the basic principles of a wheelset and leads to phenomena such as power transmission to the wheels, running safety, wear in the wheel/rail contact, and others^16–22^. This has led many researchers and scientists to develop and investigate various systems and technical solutions to improve the properties of railway vehicles with independently rotating wheels together while reducing negative effects. In principle, the height of the passengers' compartment floor in urban rail vehicles is mainly determined by the undercarriage design, including the layout of wheelsets, drivetrain, and brake system^23,24^. Tram undercarriage designs, which have adopted independently rotating wheels instead of standard wheels mounted on a common axle, are referred to as wheel kits or blocks due to their unique nature^25,26^.

**Figure 1:**
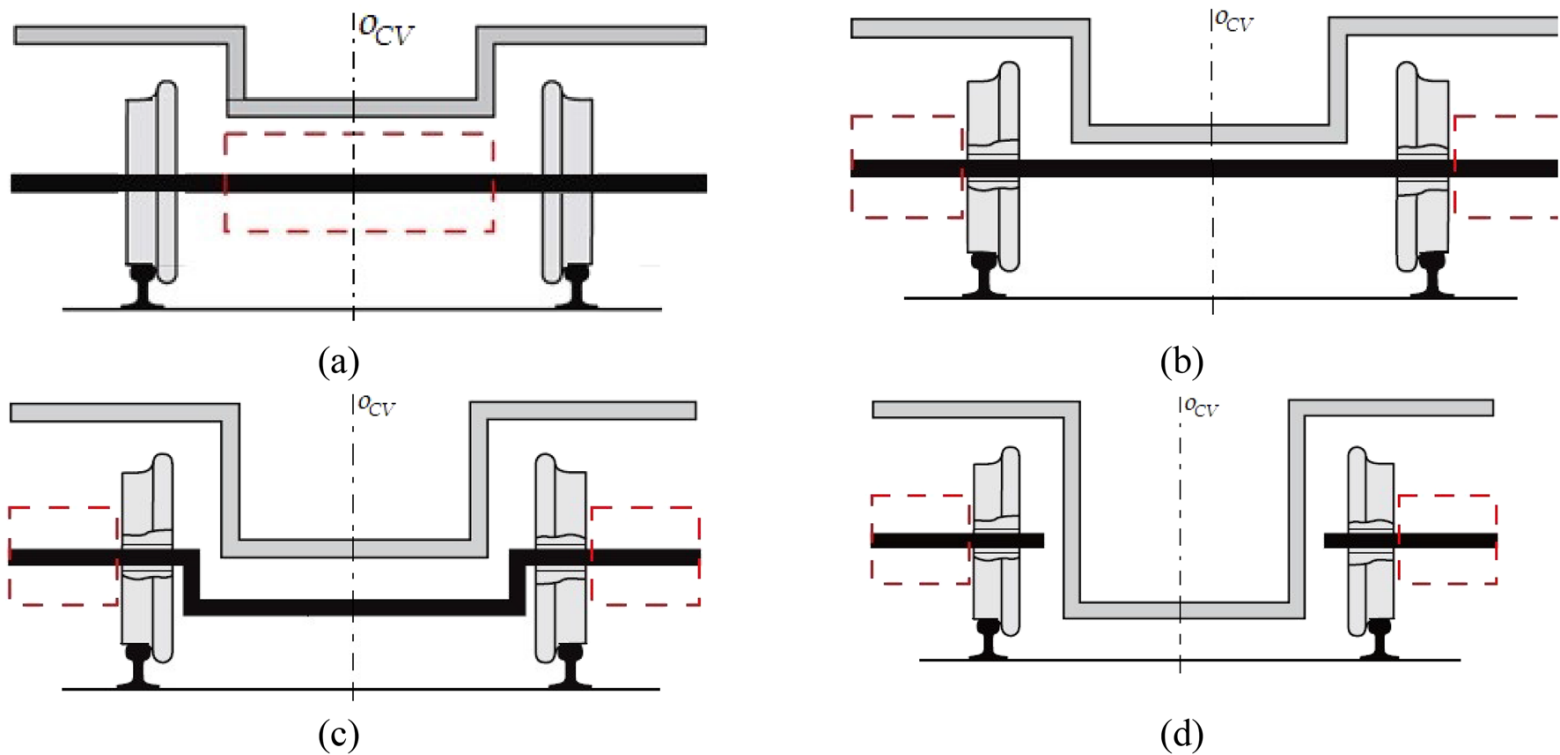
Designs of urban rail vehicles undercarriages regarding a floor height: (**a**) Wheelset with drive-train and braking system positioned between the wheels; (**b**) Wheelset with drive-train and braking system positioned outside wheels; (**c**) Undercarriage with independently rotating wheels mounted on a portal beam and external arrangement of drive-train and braking system; (**d**) Undercarriage with independently rotating wheels blocks and external arrangement of drive-train and braking system.

The undercarriage designs standard wheelset and with the IRWs can be divided into four groups based on their floor height, as depicted in Fig. 1. It illustrates the lateral plane schematics of an undercarriage. Notably, the components of these designs are symmetrically positioned with respect to the central vertical axis, *o*_*cv*_.

The design depicted in Fig. 1a represents the standard wheelsets, comprising two wheels mounted rigidly on a single axle and rotating at the same frequency. The transversely placed drive-train components, suspended on an undercarriage frame, are placed in the space between the wheels so the weight of the urban vehicle is evenly distributed on both sides. The floor height is dependent on the wheel diameter, drive-train and braking system height, as well as the deformation of the suspension system's spring. In the undercarriage design illustrated in Fig. 1b, the space between the wheels is available, allowing the components of the drive-train and braking system to be located outside of the wheels, either in front of the wheels with internal support or in front of the bearings of a wheelset with external support. Furthermore, undercarriages belonging to the group illustrated in Fig. 1c have independently rotating wheels, resulting in independent rotational speed. The wheels rotate on bearings, and the axle is replaced with a fixed portal beam. The design shown in Fig. 1d is another option that utilizes independently rotating wheels which are not connected to each other by a rigid axle. The wheel blocks are individually attached to the undercarriage frame, providing additional flexibility in choosing the floor height of the passenger compartment.

The IRWs do not have a self-steering mechanism, which can lead to excessive wear and an increased risk of derailment. However, the IRWs have excellent curving performance compared to the RWs and enable the realization of a low-floor design for urban rail vehicles, as mentioned above^27^.

## The state of the art of railway vehicles equipped with independently rotating wheels (IRWs)

The use of independently rotating wheels (IRWs) in tram undercarriages is not a new idea, and it has been the subject of many unsolved problems. Therefore, numerous research activities have been performed and are still ongoing in this field. One of the latest studies, conducted by Wang et al.^27^, focuses on the implementation of steering control in a full-scale railway vehicle equipped with IRWs and negative tread conicity. The main objective was to improve the curving performance of a railway vehicle with the IRWs by applying negative tread conicity. Wei et al. investigated^28^ the properties of a vehicle equipped with a data-driven robust controller for the active steering of its driven IRWs. Using the deep deterministic policy gradient, these researchers aimed to improve the guidance and curve-negotiation behavior of the driving system of a vehicle with IRWs. The results of their simulations demonstrated that the proposed control system was able to improve the running performance of a vehicle with IRWs. The work of Prateek et al.^29^ also merits attention. These researchers investigated the use of passive stabilization control via a gyroscopic damper to complement the active steering control of IRWs. Simulation computations revealed that the gyroscopic damper significantly improves the running stability and performance of a railway vehicle in curves. Cho and Kwak^30^ have focused on addressing the two main disadvantages of the IRWs: insufficient guiding force and excessive wear. They have developed a new analytical model that includes a mathematical description of the IRWs wheelset dynamics. The resulting equations of motion can be algorithmized and used for investigating various phenomena related to IRWs. The research conducted by the authors^31^ was focused on the dynamics of railway vehicles with IRWs. The study revealed that the longitudinal creep forces of the IRWs disappear when the wheels are separated. To conduct this research, the railway vehicle was equipped with a planetary gear differential as a passive control device for the left and right wheels. The researchers Lu et al.^32^ have investigated methods for reducing wear and improving the guidance of independently rotating wheels (IRWs). The research was carried out through co-simulations in Matlab and Simpack software. The results demonstrated that the applied control method improved the vehicle's running performance and reduced wear of both the wheel and rail.

In principle, the selected studies discussed in this review, as well as many others^14,33–38^, address the two primary issues of IRWs, namely the excessive wear of the wheel/rail couple and an indefinite movement of a wheelset or a vehicle. These findings have prompted the researchers to ask two questions, whether it is possible to design an alternative technical solution for the wheelset to improvea railway vehicle's performance while it is curving and what the energy efficiency of such a technical solution is.

Energy consumption is an important indicator of the energy efficiency of railway vehicles. It should be noted that the use of innovative constructions of urban rail vehicle undercarriages is one way to create energy-efficient railway vehicles. Promising approaches in this direction include means for controlling the installation of undercarriages and wheelsets on the rail track^39–41^, as well as the use of independently rotating wheels^42–45^.

The research team has developed the idea of a perspective constructive scheme for a railway wheel, abbreviated as the PKS wheel. The concept behind this technical solution is that the guiding surface, i.e., the flange, rotates independently on the guiding surface, i.e. the flange, rotates independently to the tread surface of the wheel instead of the entire wheel (i.e. one monoblock of the tread surface and the flange) which rotates around the axle axis. There are several ways to achieve this^46^.

To investigate the effects of the PKS wheel design on running resistance, various studies have been conducted. These studies have revealed that the PKS wheel design has a positive impact not only on the wear of the wheel/rail surfaces and noise reduction but also on the overall running safety of the railway vehicle^47^.

However, the effectiveness of known measures for implementing passive corneringundercarriages or wheelsets in curved sections of the track has proven to be insufficient^42,54^. The forced control of undercarriage installation in the rail track requires the use of complex and expensive control systems^39,48,49^. Furthermore, the use of independently rotating wheels has resulted in problems with centring wheelsets in the rail track, increased wear on flanges, as well as increased wear on rail surfaces^50,51^, and has also affected traffic safety by increasing the risk of wheels climbing off the rails. This has led to an increase in the level of kinematic running resistance of rail vehicles^52–54^.

According to works^47,55^, a novel constructive scheme for railway wheels, referred to as PKS, was proposed and justified to address the aforementioned issues. The PKS wheel design differs from the traditional IRW design, as it allows for independent rotation of the supporting surface, or wheel tread, from its guiding surface, or flange. This technical solution can potentially decrease the amount of kinematic running resistance of railway vehicles and enhance their safety during movement, in comparison to traditional TKS wheel designs.

**Figure 2:**
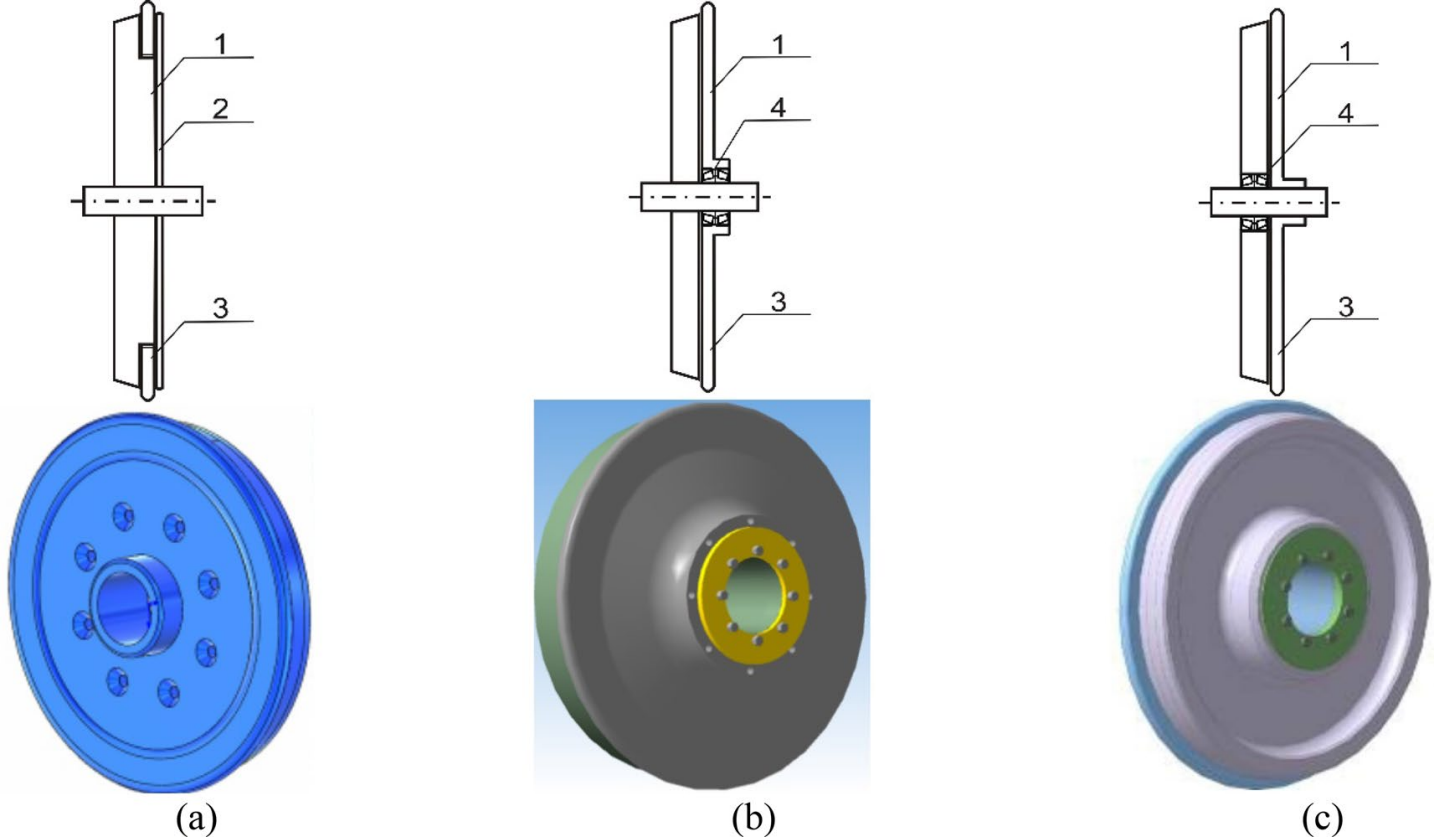
The designed PKS railway wheel: (**a**) the PKS wheel Variant 1; (**b**) the PKS wheel Variant 2; (**c**) the PKS wheel Variant 3; 1—a wheel hub, 2—a pressing disc; 3—a flange, 4—a bearing.

Three different designs of the PKS wheel were developed^63,64^, which are depicted in Fig. 2 along with their 3D models. These designs are referred to as Variant 1, Variant 2, and Variant 3.

These designs can be categorized into three groups based on the interaction between the supporting and guiding surfaces of the wheel with each other and the wheel axis.

•Variant 1—a rigidly fixed supporting surface on the wheel axle and a guiding surface (the flange) mounted on the supporting surface with independent rotation (Figure 2a);

•Variant 2—the supporting surface is also rigidly fixed on the wheel axle, but the guiding surface is mounted on the wheel axle with independent rotation (Figure 2b);

•Variant 3—the guiding surface (the flange)is rigidly fixed on the wheel axle, and the supporting surface is mounted on the wheel axle with independent rotation (Figure 2c).

The technical solution of a railway wheel design, namely the PKS wheel, is primarily applicable to railway vehicles operating at lower speeds and is particularly effective in navigating small track curves. Trams, being a type of railway vehicle, are thus the most suitable for the installation of these wheels. It is noteworthy that the PKS wheels can be successfully integrated with the low floor concept, which employs independently rotating wheels.

The primary objective and expected contribution of this research are to explore and evaluate the impact of the PKS to the kinematic running resistance and energy efficiency. This includes:

- High energy efficiency of railway vehicles operation;
- High ride comfort included low vibrations, low noise, and smooth movement of railway vehicles^47,56^;
- Low maintenance cost of both railway vehicles and needed infrastructure;
- Reliable long-term operations.

Kinematic running resistance is a crucial factor contributing to the total running resistance of a railway vehicle, which determines the energy consumption during a movement of a transport unit. The running resistance is an essential performance indicator that reflects the energy efficiency of a railway vehicle, comprising primary and additional movement resistances.

Components of the main movement resistance are resistances due to friction in bearings ^57–59^, the friction of rolling and sliding of wheels on rails, the dissipation of energy to a track and into the environment, and aerodynamic resistance.

The magnitudes of most of the components of the rail vehicle's running resistance practically do not depend on the design of its undercarriages, and it is difficult or impossible to purposefully influence these values. Having determined and compared the mechanical energy costs arising due to the work of creep forces in the wheel/rail contact during the movement of variants of undercarriages of an urban rail vehicle, it is possible to choose the best variant of its constructive scheme. As a result, it is possible in this way to influence the level of kinematic running resistance of an urban rail vehicle.

During the rotation of a railway wheel, elastic sliding along the rail occurs, in addition to rolling friction. This phenomenon is characterized by the dimensionless coefficient of elastic sliding, also known as creep, which represents the deviation from the conditions of pure rolling. Moreover, in certain circumstances, non-elastic sliding of the rolling surfaces of the wheels and ridges along the rails may also occur.

The main contributors to the resistance of a railway vehicle's movement on a track are associated with the forces generated by the wheel slipping on the rails, both in straight and curved sections of a railway track. The kinematic running resistance is thus linked to the work of creep forces at the wheel/rail contact points, which ultimately leads to the dissipation of mechanical energy during railway vehicle movement. The magnitude of kinematic running resistance is influenced by numerous factors, including the radii and lengths of curves, the implementation of measures to lubricate the contact surfaces of wheels and rails, the technical conditions of railway vehicles and tracks, as well as the features of their structural device.

Incorporating PKS wheels in the undercarriage of urban rail vehicles facilitate a notable reduction in their primary running resistance by minimizing the sliding component of the wheels on the rails. Moreover, the use of PKS wheels partially reduces the additional resistance encountered by railway vehicles when moving along curved sections of a track.

## Simulation analysis of railway wheel designs on kinematic running resistance

The main objective of this presented study is to evaluate the reduction of consumption of mechanical energy for these components during the movement of an urban rail vehicle undercarriage with various design options for the wheels.

In addition, the characteristics of the rail vehicle movement on a track are affected by many random factors, which causes certain difficulties in determining the possible effect of the use of certain technical solutions in undercarriages from the standpoint of influence on the level of kinematic running resistance. Therefore, when conducting research on the influence of the design features of the undercarriages of urban rail vehicles on the level of kinematic running resistance of their movement, it is appropriate to determine the characteristics of mechanical energy losses with reference to a certain route of movement.

**Figure 3:**
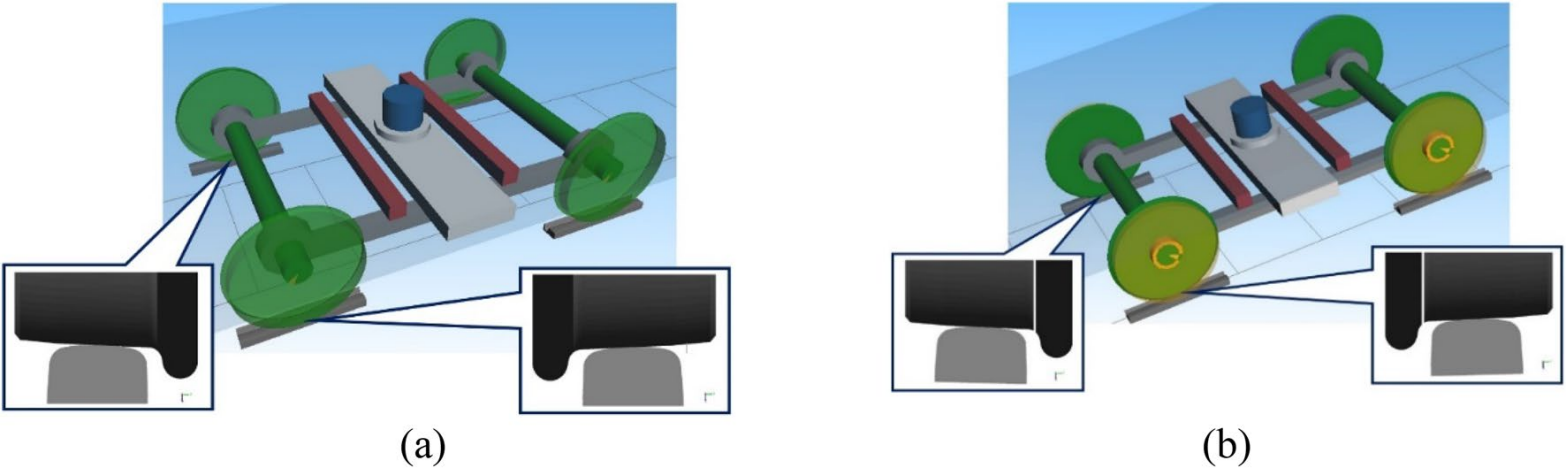
Multibody models of the analyzed urban rail vehicle undercarriage with (**a**) Version 1—with the TKS wheels; (**b**) Version 2—with the PKS wheels.

To determine the influence of the use of PKS wheels in an undercarriage of urban rail vehicles on the level of their kinematic running resistance, simulations of the movement of a Tatra tram, a T3 model with two versions of the undercarriages have been carried out (Fig. 3):

- Version 1—the undercarriage with the TKS wheels;
- Version 2—the undercarriage with the PKS wheels.

Multibody models of these undercarriages were created using Simpack software, which is widely utilized by researchers and scholars for evaluating the running properties of railway vehicles^60,61^. The simulation computations were conducted in two stages. In the first stage, the creep forces and slip velocity were evaluated for two versions of the urban railway vehicle undercarriage (Version 1, Version 2) in curved sections of a rail track with varying radii and running speeds. The second stage of the simulation was performed to assess the average power for both versions of the railway vehicle undercarriage (Version 1, Version 2).

It should be noted that there are no features or differences in the design of the urban rail vehicle undercarriages with the different types of wheel designs. All components of both undercarriages are the same along with all guiding and supporting properties. The wheels with the TKS as well as with the PKS are replaceable. The main difference between the undercarriages shown in Fig. 3 consists in the presence of additional degrees of freedom in the undercarriage with the PKS wheels design (Fig. 3b) due to the wheel design described above.

The performed simulation analyses include movements of the undercarriage with the TKS wheel design and with the PKS design on a track section in a curve. The description of the used simulation multibody model (parameters of the undercarriage and the track) is in Description of the multibody model used in the study.

The evaluation of the outputs can be recognized in two stages.

In the first stage of the analysis, the two indicators characterizing the interaction of the wheels with the rails have been calculated and compared. They are as follows:

- The creep force components (*T*_*x*_, *T*_*y*_) in the wheel/rail contact points;
- The slip velocity components (*v*_*x*_, *v*_*y*_) in the wheel/rail contact points.

The knowledge of these indicators’ values enables the determination of the amount of mechanical energy loss resulting from the work of creep forces. These losses determine the magnitude of the kinematic resistance to the movement of the rail vehicle. Analyzing the obtained values allows for the comparison of the kinematic resistance characteristics of different variants of the tram vehicle based on the radius of the curve and the speed of movement. The results of the first stage of the research are presented in Findings from the initial stage of the study: creep forces and slip velocity in the wheel/rail contact and discussed in Discussion of the study's results.

The second stage of this research includes calculation and a comparison of the following output:

- The average power ($\overline{N}$) dissipated in the wheel/rail contact points during the movement of two versions of the undercarriage of an urban railway vehicle

Thus, the second stage of simulation computations has been carried out to determine the impact of the change of the wheel constructive schemes on the level of kinematic running resistance of urban rail vehicles. This included comparative computations to determine the loss of mechanical energy due to the work creep forces in the wheel/rail contact points in the movement of these vehicles on a particular railway track. According to the applied methodology, the mechanical energy consumption in the wheel/rail contact points of vehicles has been computed based on the determination and summation of the mechanical work of each component of the creep forces in each wheel/rail contact of an urban rail vehicle.

The mechanical energy *E*_*abi*_ dissipated in each contact when wheels slide along the rails is equivalent to the work of the creep forces *A*_*abi*_ in this contact. It has been defined as the sum of the products of the components of the creep forces in the corresponding wheel/rail contact and slips relative to the rail at the contact point in this direction, taken by module, as given in Eq. (1):

1 $$A_{abi} = \left| {T_{abi}^{x} \cdot S_{abi}^{x} } \right| + \left| {T_{abi}^{y} \cdot S_{abi}^{y}} \right|$$

where *a*, *b* indicate the number of the wheelset of a vehicle in the running direction and a side of the urban rail vehicle (left or right), respectively, $T_{abi}^{x}$ and $T_{abi}^{y}$ are the longitudinal and lateral components of the creep force in the *i*-th wheel/rail contact and $S_{abi}^x$ and $S_{abi}^{y}$ mean slippage in the *i*-th wheel/rail contact of the corresponding wheel in the longitudinal and lateral directions, respectively.

Taking into account the rapid change of the values of the creep forces in the corresponding wheel/rail contacts, consideration was given to their values when modelling for a specific sampling frequency *f*. Then, slippage was computed based on the components of the slippage speed in each contact based on the following formula (2):

2 $$\begin{gathered} S_{abi}^{x} = v_{{x_{abi} }} \cdot t \hfill \\ S_{abi}^{y} = v_{{y_{abi} }} \cdot t \hfill \\ \end{gathered}$$

where $v_{{x_{abi} }}$ and $v_{{y_{abi} }}$ are the components of the sliding speed in each contact in the longitudinal and lateral directions, respectively, and *t* is the simulation period taking into account the sampling frequency *f* according to Eq. (3):

3 $$t = \frac{1}{f}$$

The total expenditure of mechanical energy due to the sliding in the wheel/rail contact points during the vehicle running equals the sum of the work of the creep forces during sliding in each wheel/rail contact. It is computed by Eq. (4):

4 $$A_{\Sigma } = \sum\limits_{a = 1}^{4} {\sum\limits_{b = 1}^{2} {\sum\limits_{i = 1}^{2} {A_{abi} } } }$$

Accordingly, the average power dissipated during the wheels sliding on the rails when the vehicle runs on the route was determined according to the Eq. (5):

5 $$\overline{N} = \frac{{A_{\Sigma } }}{{t_{total} }}$$

where *t*_*total*_ is the total time of the vehicle's movement on the considered route.

At the second stage of the simulation, studies have been carried out on the influence of the use of the PKS wheels in the undercarriage of the urban rail vehicle on the level of kinematic running resistance when it runs on the chosen railway track section.

The results of the research in the second stage are presented in Findings from the second stage of the study: average power in the wheel/rail contact and discussed in Discussion of the study's results.

### Description of the multibody model used in the study

Regarding a multibody model of the undercarriages, it consists of rigid bodies interconnected using flexible elements, such as springs, dampers and other kinematic and mechanical joints. These models have considered all real allowed movements of individual bodies and other specifics of these virtual models^62,63^. The created multibody models of the undercarriage with the TKS wheels and with the PKS wheels are shown in Fig. 3.

It should be noted that the research team has developed more than one models of an urban rail vehicle undercarriage with the PKS. These models and proposals have been published, e.g. in^47,56^. In principle, multibody models come from three designs of the PKS of wheels, which are depicted in Fig. 3. The model created in the Simpack software and presented in Fig. 3b is equipped with the wheelsets according to Fig. 2b.

The Fig. 3 shows two undercarriage multibody models, particularly, the undercarriage with the TKS wheels design (Fig. 3a) and the undercarriage with the PKS wheels design (Fig. 3b). The difference between these two models is only that the undercarriage in Fig. 3b has additional degrees of freedom for the rotation of flanges around the axis of rotation (with four additional degrees of freedom).

**Figure 4:**
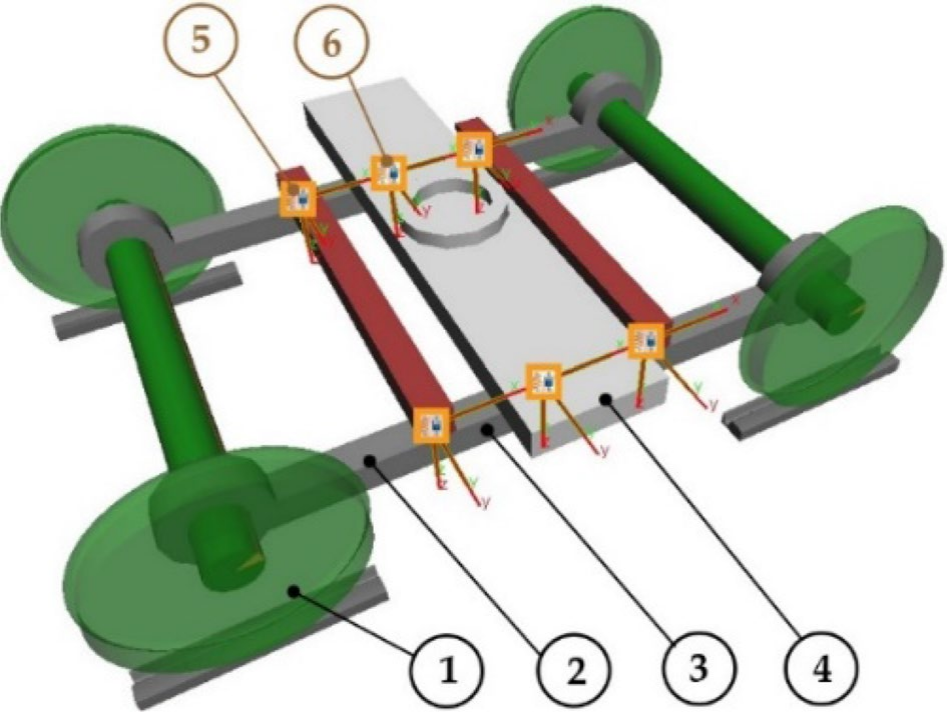
Main components of the undercarriage: wheelsets (1), a frame (2), lateral beams (3), a crossbar (4), and suspension elements (5 and 6).

The multibody model of the undercarriage consists of rigid bodies interconnected by flexible massless elements and by mechanical couplings. There are the following bodies (Fig. 4): wheelsets (1), a frame (2), lateral beams (3), a crossbar (4). These bodies are connected by means of force elements (5 and 6), which form a suspension system of the undercarriage. The selected parameters of the bodies of the modelled undercarriage are listed in Table 1.

**Table 1:** Parameters of main rigid bodies of the undercarriage model.

Component	Mass [kg]
Wheelset	380
Frame	1100
Lateral beam	220
Crossbar	450

The study has taken into account that the undercarriage of the vehicle is subject to a load equivalent to half the vehicle's body mass of 23 tons, applied at a center pivot by the body crossbar. The wheel utilized in the experiment featured the E-99–00 profile^64^ and a diameter of 0.68 m. Additionally, the undercarriage had a wheelbase of 1.9 m. The suspension system comprised two sets of flexible elements; the first set was positioned between the frame and the lateral beams with a vertical stiffness of 1,200,000 N/m. The second set with a vertical system of 510,000 N/m connected the crossbar and the lateral beams.

The model has been running on a railway track with the wide track gauge and the B1 rail profile. Simulation computations have been performed on the model track with the curve radii of *R* = 20 m, 50 m, 100 m, 150 m, 200 m and 250 m at the running speeds of *v* = 10 km/h, 20 km/h, 30 km/h and 40 km/h. The sampling rate has been set at the value of 250 Hz.

In the Simpack program, the contact between the wheel and rail is defined using available modeling elements. These modeling elements include the definition of the rail head profile and the wheel running surface. As for the rail profile, the B1 profile^65^ was used, and its corresponding coordinates were provided for both the right and left rails separately. Rail description details are prescribed in the modeling element Rails, which communicates directly with the modeling element Rail-Wheel Pair. The rail has no defined inclination; therefore, the rail cant is equal to zero, and the rediscretization step is set to a value of 0.0005.

**Figure 5:**
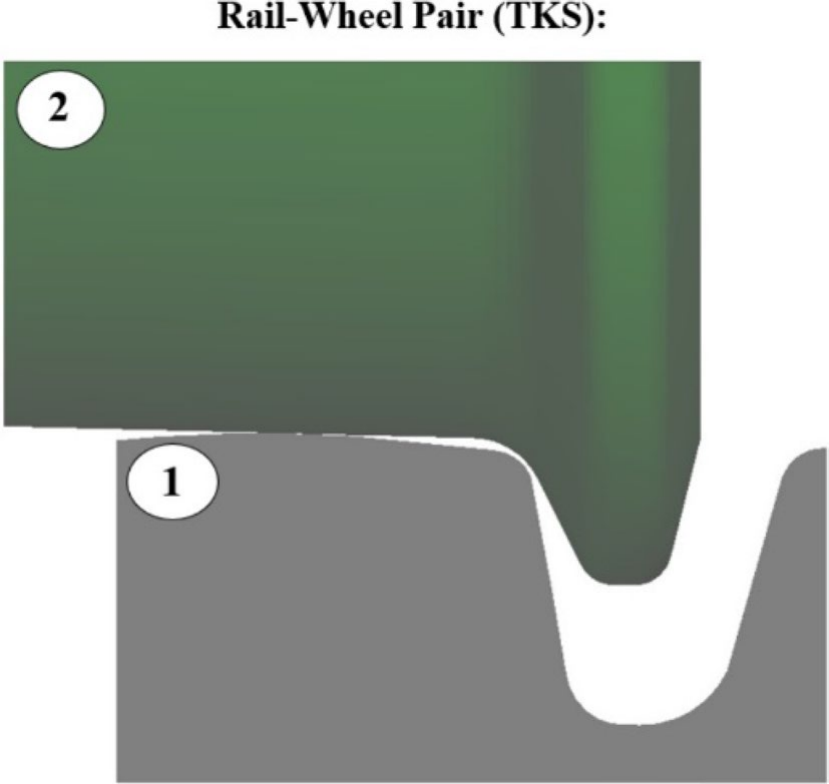
Components of the Rail-Wheel Pair of the TKS: 1—the rail, 2—the wheel.

The wheel models vary depending on whether they are designed for trams with TKS wheels or PKS wheels. In the TKS wheel model of an electric tram, the wheel profile is defined in a single source file that contains the required coordinates for the wheel's running surface and flange profile. The E-99–00 profile is utilized. As a result, the process adheres to the standard procedure found in other railway vehicle models with monobloc wheels. The Rail-Wheel Pair includes two separate bodies, i.e. the wheel and the rail (Fig. 5).

**Figure 6:**
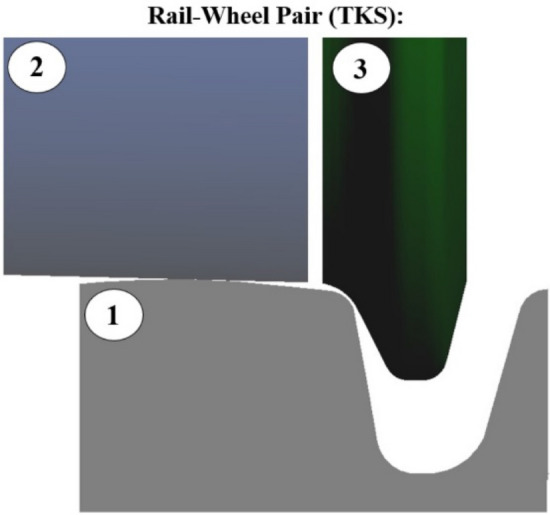
Components of the Rail-Wheel Pair of the PKS: 1—the rail, 2—the rotating running surface of the wheel, 3—the rotating flange.

In the case of the PKS wheel, the approach to the wheel model is different. Two separate modeling elements, Rail-Wheel Pair, are required: one for the wheel's running surface and the other for the flange profile. The running surface is represented by a single rotating body (body 2) (Fig. 6), while the flange profile is represented by the second, independently rotating body (body 3). The flange profile is defined in a separate file and corresponds to the E-99–00 profile, divided into two separate parts.

The schemes of the "Rail-Wheel Pair" modeling element for TKS and PKS wheels are shown in Fig. 5 and Fig. 6. As evident from the scheme, two "Rail-Wheel Pair" elements had to be defined for the PKS wheel; one for the wheel/rail contact pair and the other for the flange/rail contact pair. As a result, the simulation calculations for the electric tram with PKS wheels required a longer computation time.

The contact model of the wheel and rail is defined in the used program by the Rail-Wheel Contact element. It is important to ensure the correct choice of parameters for calculating normal and tangential forces. In both the TKS and PKS models, the same calculation parameters were specified.

The following parameters were used to calculate the normal forces in the contact: wheel rediscretization steps 0.0005 m, Young's modulus 2.1.1011 Pa, Poisson's ratio 0.28, and contact reference damping 100 kNs/m.

The calculation of tangential forces in the contact is performed using the FASTSIM method. The parameters used were: Kalker weighting factor 1, minimum reference velocity 0.01 m/s, friction coefficient 0.25, stick friction coefficient 0.4, stiffness for the stick 100.106 N/m, and damping for the stick 1000 Ns/m.

As it is mentioned above, there are many various researchers focused on the investigation of the properties of the wheelsets with the IRWs ^28,66,67^. However, only a few works are available with the research of the railway wheelsets design with the technical solution including the independent rotating flange of a railway wheel.

Therefore, the main novelty of the presented research is the analysis of the operational properties of an undercarriage equipped by the PKS. Specifically, it is an investigation of the influence of this technical solution to the topical characteristics of every transport means, i.e. energy efficiency. In this case, it is the evaluation of kinematic running resistance. If the kinematic running resistance could be reduced, it would lead to the more effective operation of urban railway vehicles.

The awaited outputs were obtained based on specific sensors of output quantities. These sensors have been defined mainly in the wheel/rail contacts for obtaining the force values.

### Ethical approval

This work was prepared according to the rules of good practice.

## Research results

### Findings from the initial stage of the study: creep forces and slip velocity in the wheel/rail contact

**Figure 7:**
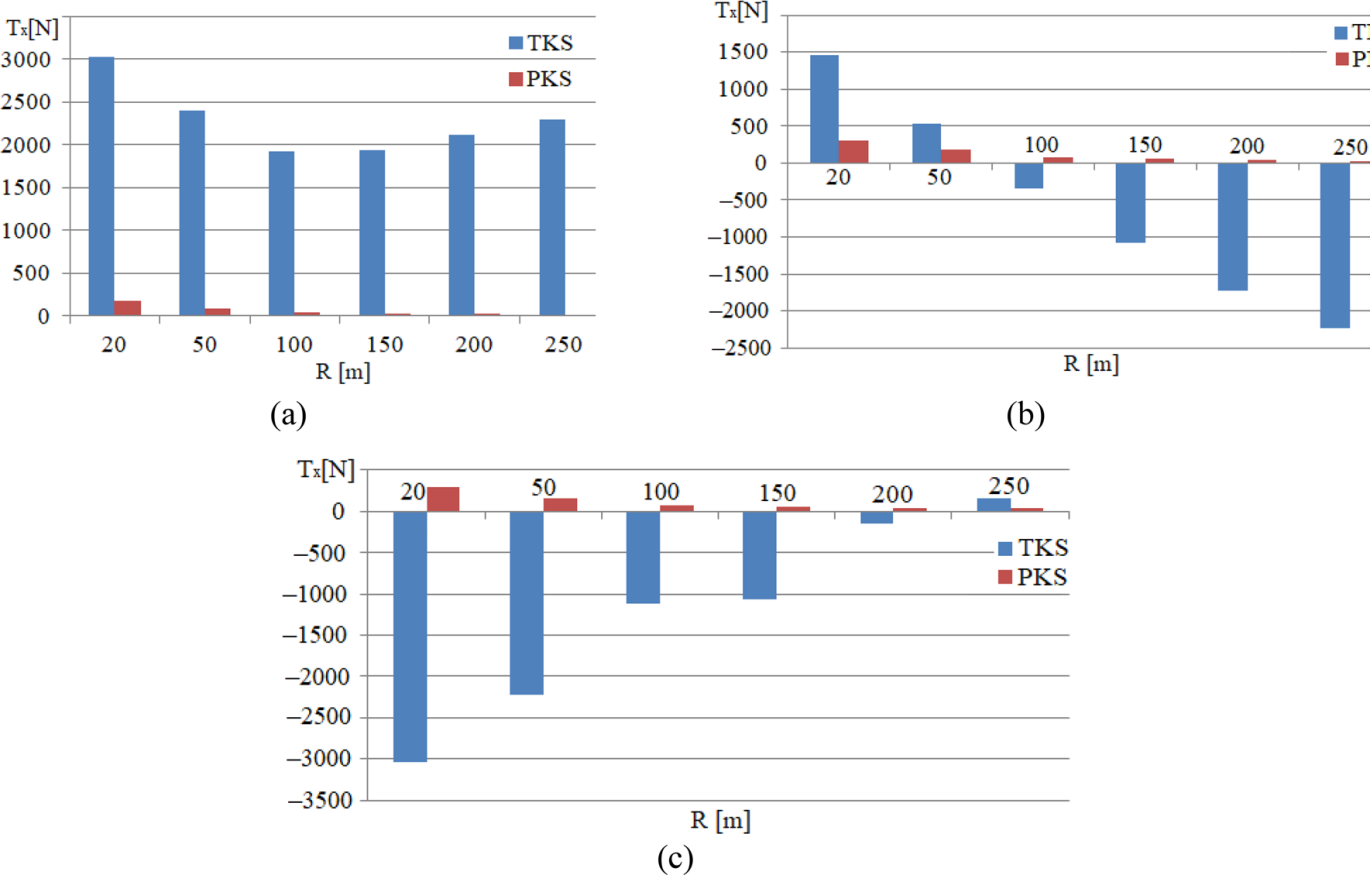
Graphs of the dependence of the *T*_*x*_ value on the curve radius at the running speed of *v* = 10 km/h: (**a**) For a contact point with the rail of the supporting surface of the attacking wheel; (**b**) For a contact point with the rail of the guiding surface of the attacking wheel; (**c**) For a contact point with the rail of the supporting surface.

**Figure 8:**
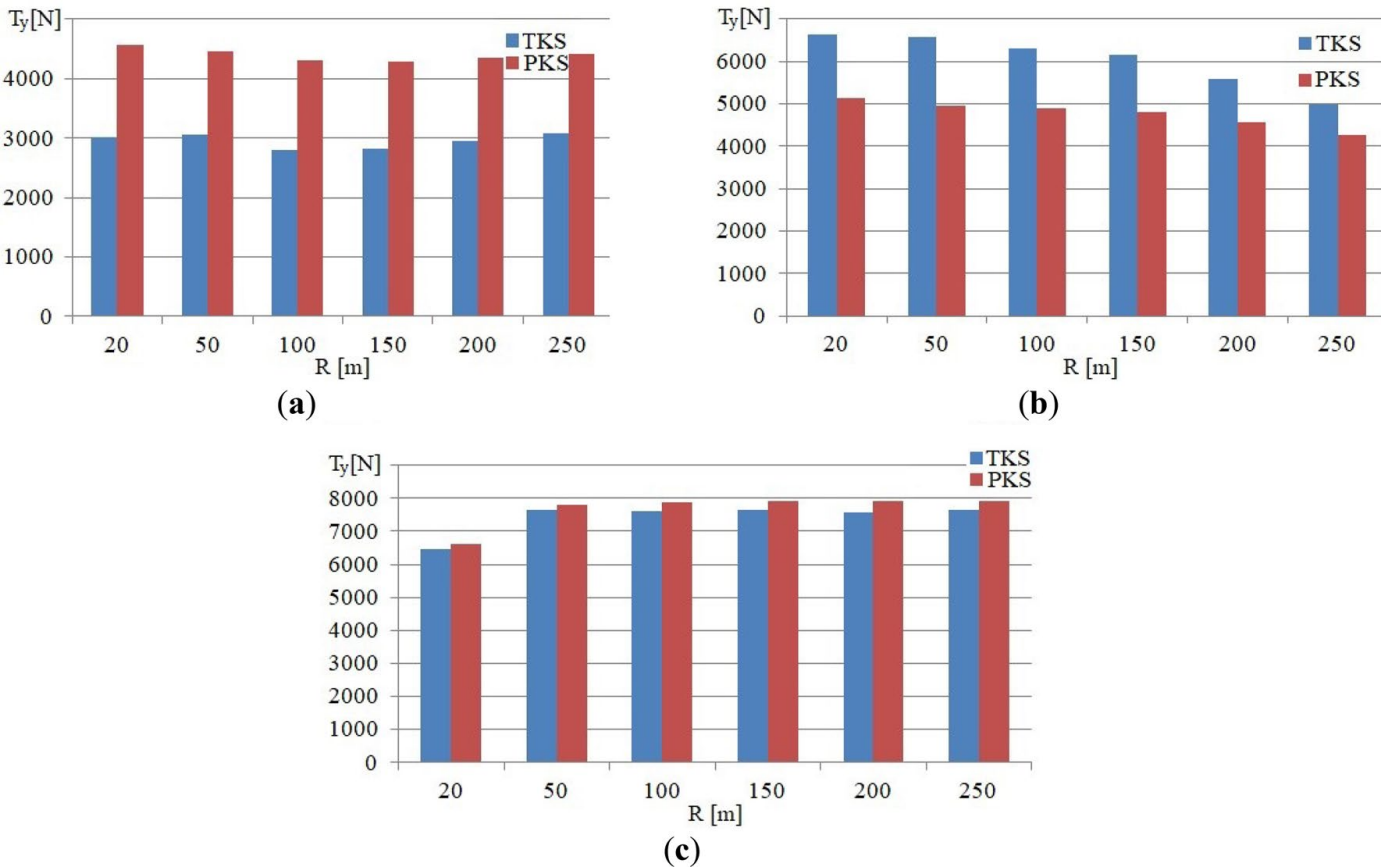
Graphs of the dependence of the *T*_*y*_ value on the curve radius at the running speed of *v* = 10 km/h: (**a**) For a contact point with the rail of the supporting surface of the attacking wheel; (**b**) For a contact point with the rail of the guiding surface of the attacking wheel; (**c**) For a contact point with the rail of the supporting surface of the non-attacking wheel.

**Figure 9:**
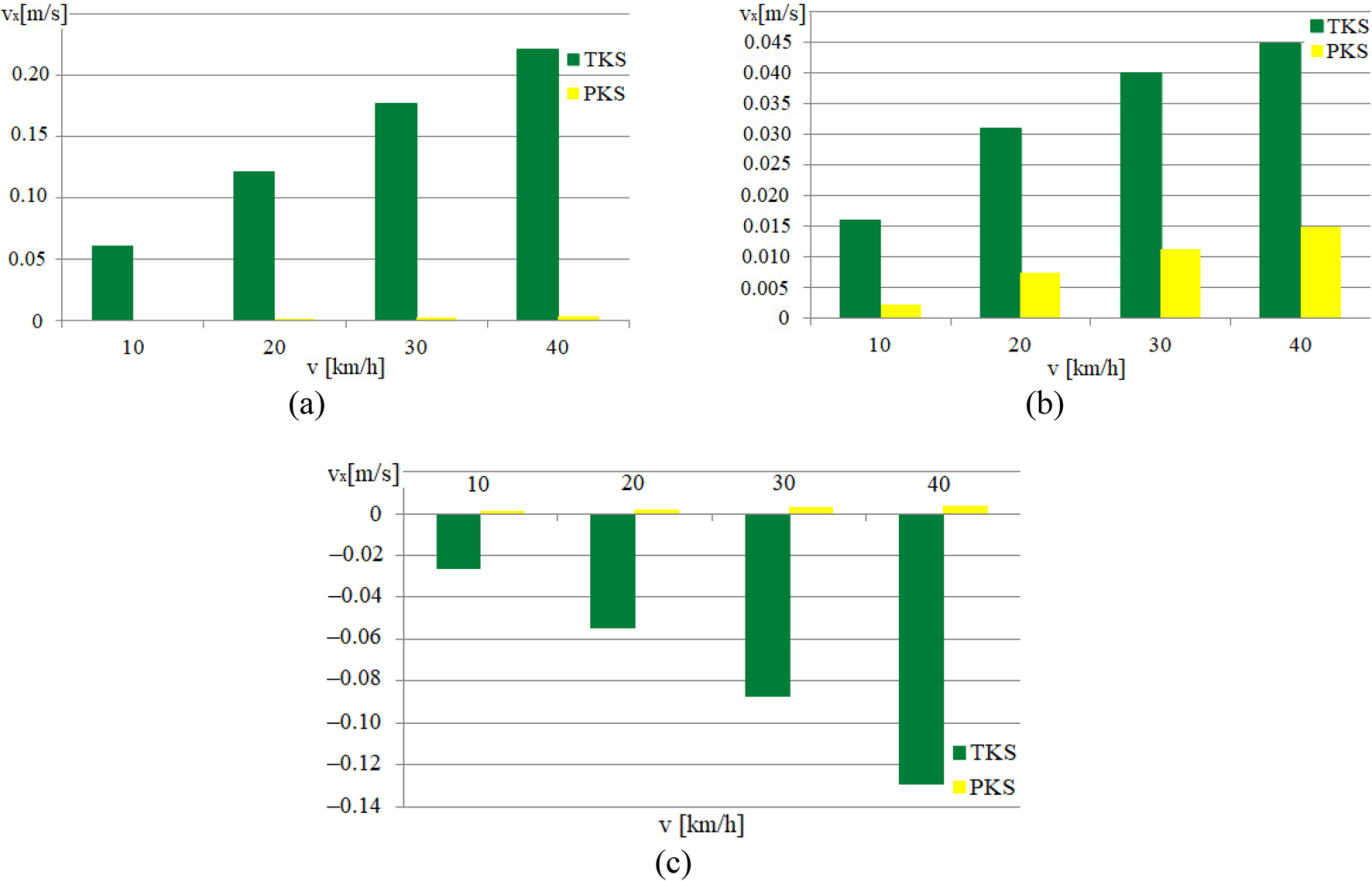
Graphs of the dependence of the value of *v*_*x*_ on the running speed in the curve with R = 50 m (for the front wheelset): (**a**) For a contact point with the rail of the supporting surface of the attacking wheel; (**b**) For a contact point with the rail of the guiding surface of the attacking wheel; (**c**) For a contact point with the rail of the supporting surface of the non-attacking wheel.

**Figure 10:**
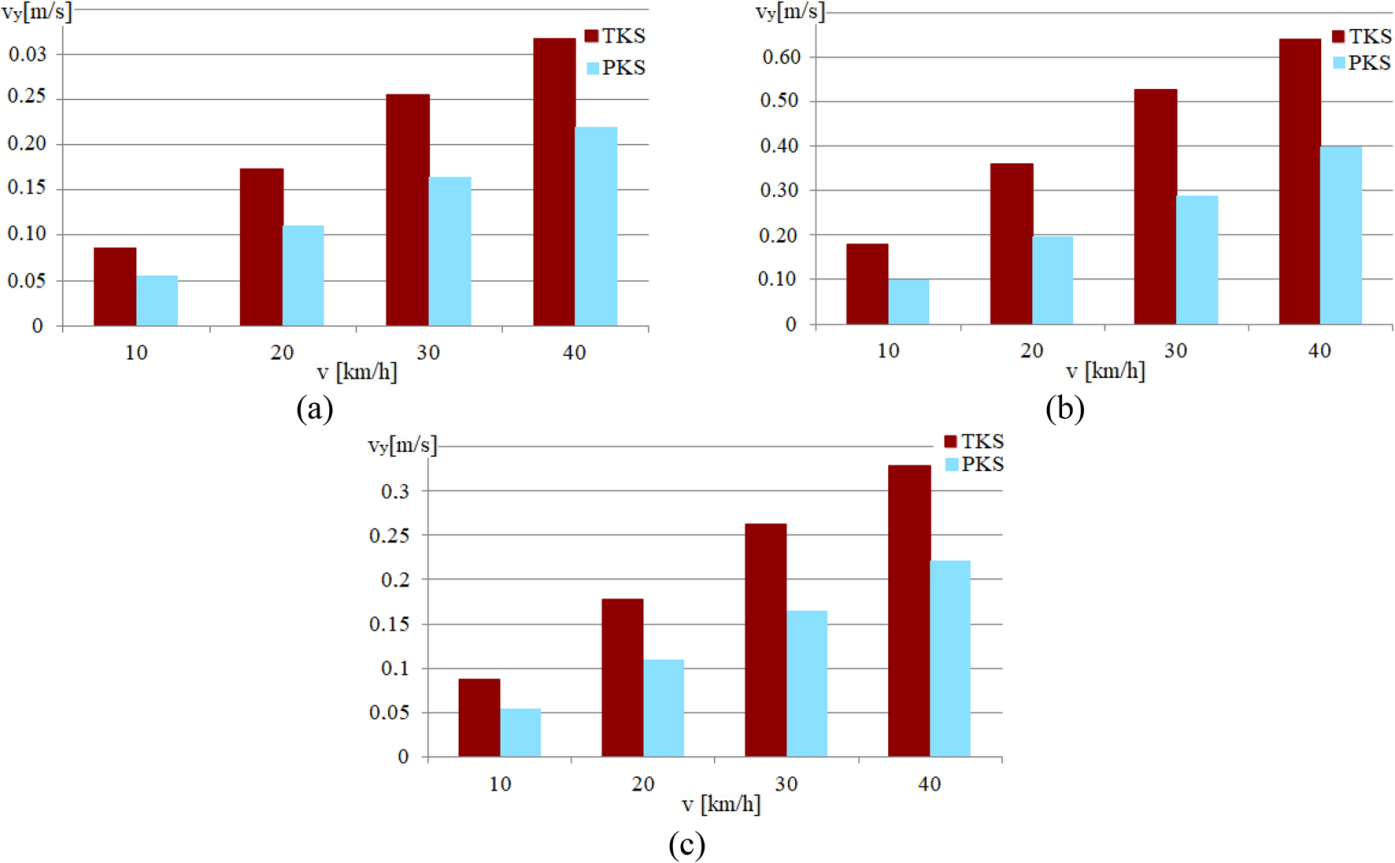
Graphs of the dependence of the value of *v*_*y*_ on the running speed in the curve of *R* = 50 m (for the front wheelset): (**a**) For the contact point of the bearing surface of the attacking wheel with the rail; (**b**) For the contact point of the guiding surface of the attacking wheel with the rail; (**c**) For the contact point of the bearing surface of the non-attacking wheel with the rail.

**Figure 11:**
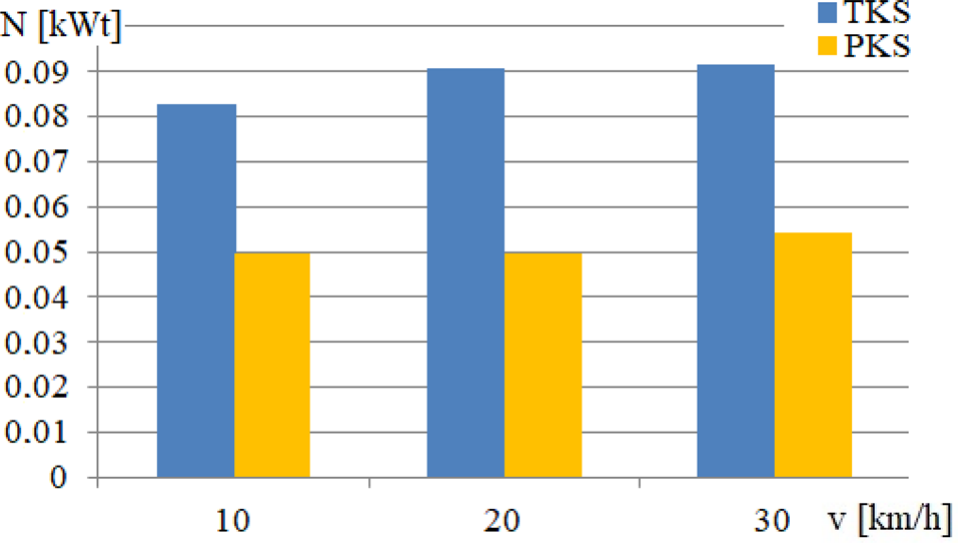
Dependences of the average power values on the running speed.

The Figs. 7,8,9,10,11 present obtained results of the computation of the indicators for Version 1 (TKS) and Version 2 (PKS) of the urban railway vehicle undercarriage.

Then, Fig. 7 shows graphs of the dependence of the magnitude of the longitudinal component of the creep forces *T*_*x*_ at the points of contact with the rails of the supporting and guiding surfaces of the attacking wheel and the support surface of the non-attacking wheel of the front wheelset on the curve radius. They are computed at the running speed of *v* = 10 km/h and for running in curves with various radii.

The Fig. 7 shows graphs of similar dependences of the lateral component of the creep forces *T*_*y*_ at the contact point during the urban rail vehicle undercarriage running in curves with various radii and at the same running speed of *v* = 10 km/h.

The Fig. 8 shows graphs of the dependences of the lateral component of the creep forces *T*_*y*_ at the corresponding contact points with the rails of the supporting surface of the attacking wheel when the urban rail vehicle undercarriage moves in curves with various radii at the speed of 10 km/h.

The Fig. 9 illustrates that the PKS wheel design reduces the *v*_*x*_ value for all investigated running speeds compared to the TKS wheel design. This reduction leads to significant savings in mechanical energy and enhances the overall efficiency of the railway vehicle equipped with PKS wheels.

The Fig. 10 shows a comparison of the slip velocities *v*_*y*_ for the TKS and PKS wheel designs. It can be seen that the value of *v*_*y*_ has an increasing tendency depending on running speed. It is obvious, that the PKS wheel design leads to a reduction in the value of the *v*_*y*_ for all three evaluated causes.

### Findings from the second stage of the study: average power in the wheel/rail contact

The Fig. 11 shows an example of the dependence of the average power on the running speed, which is dissipated when the tram wheels slide on the rails of the chosen railway track section. Therefore, an analysis of the obtained results from simulation computations allows concluding that the application of the PKS wheels as a component of an urban rail vehicle undercarriage led to a significant reduction significantly (up to 40%) in the value of the kinematic running resistance of its movement and the loss of mechanical energy, which is dissipated in the wheel/rail contacts.

Thus, it can be seen in Fig. 11, that the value of the average power for the undercarriage with the TKS wheels and for the running speed of 10 km/h is 0.0835 kWt. When this undercarriage runs at the speed of 20 km/h, the average power increases to the value of 0.091 kWt. However, if the same undercarriage runs at the speed of 30 km/h, the value of the average power does not increase so significantly and the achieved value is 0.0922 kWt.

On the other hand, the installation of PKS wheels significantly contributes to the reduction of the average power value for all investigated running speeds. The average power value remains consistent for running speeds of 10 km/h and 20 km/h, hovering around 0.05 kWt. However, for a running speed of 30 km/h, the value slightly increases to 0.055 kWt. The use of PKS wheels in railway vehicles offers clear advantages due to this reduction. The percentage reduction in the average power for the running speed of 10 km/h is 60%, 55% for 20 km/h, and 59.7% for 30 km/h.

## Discussion of the study's results

It is known that the kinematic running resistance of a railway vehicle and the corresponding amount of mechanical energy that is lost when the wheels slide on the rails can be quantified by the amount of work (or power) of the creep forces. Therefore, at the initial stage of the research, a simulation of the movement of variants of the urban rail vehicle undercarriages in curved sections of the track at different running speeds has been carried out. By means of force sensors in the wheel/rail contact, signals of creep forces have been obtained from simulation computations. The values of indicators affecting the level of operation of the creep forces were studied and compared, namely the values of the longitudinal *T*_*x*_ and the lateral *T*_*y*_ components of the creep forces in the wheel/rail in the supporting surfaces and the guiding surfaces of the attacking wheel and the support surface of the non-attacking wheel of the front wheel pairs, as well as values of the component slip velocities *v*_*x*_ and *v*_*y*_ in the corresponding contacts. The angle of attack of the front wheelset on the rail while navigating curved sections of the track has also been calculated.

The analysis of the obtained computation results shows that, for the accepted running conditions, the magnitudes of the longitudinal component of the force of the bulwark *T*_*x*_ at the contact points with the rails of the supporting surface of the attacking wheel for the PKS variant are insignificant (the values of 30 N to 50 N), while the values of the *T*_*x*_ force component for the TKS variant when the undercarriage runs in curves of different curve radii have reached the values of 2000 N to 3000 N, as in Fig. 7a. This is determined by the features of the construction scheme of the PKS wheel, which allows independent rotation of the supporting and guiding surfaces of the wheel.

The computed values of the longitudinal component of the creep force *T*_*x*_ for contact points of contact with the rails of the guiding surface of the running wheel (Fig. 7b) when using the TKS wheels are also obtained for all curves, which have been taken into account, which are greater than the corresponding values for the PKS wheels.

The magnitudes of the longitudinal component of the creep force *T*_*x*_ for contact points with the rails of the supporting surface of the non-attacking wheel (Fig. 7c) are also insignificant (30 N to 50 N) for the PKS variant in comparison with the corresponding values for the TKS variant.

Analogous, the *T*_*x*_ values for the TKS variant when it runs in curves with various radii, vary from 100 N in the curve with a radius of 250 m to 3000 N in the curve with a radius of 20 m. It is obvious, that such a difference in the *T*_*x*_ values for the compared options is also determined by the design features of the PKS wheel.

Since the guiding surface of the non-attacking wheel of the front wheelset, which runs in a curved section of the track practically does not come into contact with the rail, the values of the component creep forces in this contact are equal to zero.

For the considered running conditions, the values of the lateral component of the creep force *T*_*y*_ in the contact points with the rails of the supporting surface of the attacking wheel for the PKS variant are about 4500 N, while the similar values of the *T*_*y*_ force component of the TKS variant when it runs in curves with the various radii are about 3000 N (Fig. 8a). These redistribution components of creep forces in the contact points with the rails of the supporting surface of the attacking wheel are determined by the features of the construction scheme of the PKS wheel, which allows independent rotation of the supporting surfaces of the wheels against the guiding surfaces of the wheels.

The ratio of the values of the lateral component of creep forces *T*_*y*_ for the contact of the guiding surface of the attacking wheel with the rail at the running speed in curves of 10 km/h is shown in Fig. 8b. By analyzing the graphs, it is obvious, that the value of this indicator for the TKS wheel, depending on the radius of the curve, exceeds the corresponding values computed for the urban rail vehicle version with PKS wheels by 800 N (curve 250 m) and up to 1700 N (curve 20 m).

The values of *T*_*y*_ in the contact of the rail and the supporting surface of the non-attacking wheel (Fig. 8c) for both analyzed variants of the vehicle are close to each other and the value 8000 N is reached for the most curve radii. A slight excess of the value of this parameter for the PKS wheel (8000 N) in comparison with the TKS wheel (6200 N) is in the curve with a radius of 20 m.

The obtained graphs and the ratio of *T*_*x*_ values for a vehicle equipped with TKS and PKS wheels while moving at different speeds in the considered curves demonstrate similar characteristics. This is attributed to the unique construction scheme of PKS wheels, which permits independent rotation of the supporting and guiding surfaces of the wheels.

The analysis of the obtained results shows, that *T*_*y*_ values for vehicles with the PKS wheels when they move at different running speeds in the curves under consideration slightly exceed the values of the corresponding indicators for the version of the vehicle with the TKS wheels. This is determined similarly to the previous statement, by the features of the construction scheme of the PKS wheels, which allows independent rotation of the support and guide surfaces of the wheels.

As the results of the computations, the values of other indicators that affect the level of mechanical energy losses during the movement of variants of the constructive schemes of trams were obtained. For example, Figs. 9 and 10 show the dependences of the values of the longitudinal *v*_*x*_ and the lateral *v*_*y*_ components of the slip velocities in the contact points with the rails of the supporting and guiding surfaces of the attacking wheel, and the supporting surface of the non-attacking wheel of the front wheelset, computed for the cases of the urban rail vehicle in the curve with a radius of 50 m at various running speeds.

Based on an analysis of the graphs presented in Fig. 9 it is possible to conclude, that the values of *v*_*x*_ for all the contacts of the PKS wheels, under consideration, are quite small and the value is less than 0.015 m/s. At the same time, the values of *v*_*x*_ for all considered contacts of the TKS wheels significantly exceed them. Thus, for example in the contact of the supporting surface of the attacking wheel, the front along the wheelset course with the rail (Fig. 9a), the values of *v*_*x*_ range from 0.06 m/s at the speed of 10 km/h to 0.22 m/s at the speed of 40 km/h.

It should also be noted that the nature of the dependence of *v*_*x*_ values on the speed of movement in curves of other radii and the ratio of these values for TKS and PKS wheels is similar.

The analysis of the graphs presented in Fig. 10 showed that the values of *v*_*y*_ for all the considered contacts of the wheels of the PKS have been obtained smaller than the similar values for the wheels of the TKS. It is characteristic that the values of *v*_*y*_ increase depending on the running speed. Thus, for example, for the contact of the supporting surface of the attacking wheel, the front wheelset along the course with the rail (Fig. 10a), the values of *v*_*y*_ for the PKS wheel are in the range from 0.05 m/s at the speed of 10 km/h to 0.22 m/s at the speed of 40 km/h. The corresponding values of this indicator for the TKS wheel are in the range from 0.08 m/s at the speed of 10 km/h to 0.32 m/s at the speed of 40 km/h. The dependence of the *v*_*y*_ values on the running speed for the contacts of the guiding surface of the attacking wheel of the front wheelset with the rail and the supporting surface of the non-attacking wheel of the front wheelset with the rail has a similar character. The nature of the dependence of the *v*_*y*_ values on the running speed in curves of other radii and the ratio of these values for the wheels of the TKS and the PKS wheel is also similar.

The analysis of the obtained results indicates also the realization of slightly smaller values of the angle of attack for running undercarriage with the PKS wheels when they move at the various speeds in the considered curves. This is also determined by the features of the construction scheme of the PKS wheels, which allows independent rotation of the supporting and guiding surfaces of the wheels. That is in the structure of the components of creep forces acting at the contact points of the wheels with the rails for the analyzed variants of tram carriages by the above-described change, there is also some change in the installation of the undercarriages on a railway track when negotiating them into a curve of a track section.

Thus, the obtained results indicate some transformation of the system of components of the creep forces acting in the wheel/rail contact for vehicles with the PKS wheels compared to vehicles with the TKS wheels. It is obvious, that the change in the configuration of the system of components of the creep forces acting in the wheel/rail contact affects the installation of a vehicle when negotiating a curve of a track section.

The use of undercarriages with independently rotating wheels is usually associated with their application in undercarriages for urban vehicles, i.e. for trams. Also, the undercarriages with the PKS of the wheels are supposed to be used in railway vehicles with the IRWs. However, as the analysis of well-known studies has shown, such as a wheelset of railway vehicles have a stronger tendency to a derailment risk. This is caused by an inability to centre such a wheelset to a track longitudinal axis. Despite that, a number of real urban rail vehicles use the IRWs in their undercarriages. The practice of their operation from a traffic safety point of view is quite satisfactory. In terms of the operational safety of the wheels with the PKS, many computational analyses have been performed, and which results are concluded in^54^. This has theoretically confirmed, that the PKS wheel has a greater reserve of resistance to derail than the PKS wheel applied for wheelsets with the IRWs. An issue of sufficient strength of the PKS wheels is solvable by a proper design and a proper choice of materials for their production. The study, which has been developed for specific input parameters has proven, that the designs of the PKS can withstand the prescribed load acting on the analysed mechanical system^53,63^.

The presented research and results belong to the study in progress, which is the subject of the scientific research of the authorship for a longer time. The objective is to reach a technical solution, which would apply to real operational conditions. For these purposes, some important activities should be performed and verified. There are mainly analyses of the undercarriage structure in terms of the distribution of stresses in a structure^68–70^, other dynamical analyses including flexible bodies and performing real tests on a test track or a chosen track section.

A real application of the proposed innovative technical solution of the railway wheel design requires plenty of other activities within future research in this area. It is mainly related to performing additional simulation analyses for various operational conditions including different running speeds, axle loads, types of undercarriages (differing by wheelsets guidance systems, types of suspensions, etc.), track geometries and others. Further, it would be desirable to subject the PKS to experimental tests, either on a test bench or on a real undercarriage.

To obtain conclusive and reliable results, it is imperative to conduct actual tests on a real vehicle equipped with PKS wheels. These tests would help verify the accuracy of the simulation computations and draw unambiguous conclusions about the feasibility of the proposed technical solution. Implementing this innovative solution in real-world applications would necessitate additional research in this domain. This would involve conducting further simulation analyses for varying operating conditions, such as different running speeds, axle loads, undercarriage types (differing by wheelset guidance systems, suspension types, etc.), track geometries, and other relevant factors. Moreover, it would be advisable to subject the PKS wheels to experimental testing, either on a test stand or a real bogie. Scaled tests on a test stand can be performed to facilitate this.

It should be noted that the authors neglected the slip in the calculation. The FASTSIM method used to calculate the wheel/rail contact, developed by J.J. Kalker, which allows for determining the forces in the contacts between the wheels of rail vehicles and the rails, is quite common and is currently widely used in the mathematical modeling of rail vehicle motion on the track.

When considering the three-dimensional contact problem between the wheel and rail, Kalker showed that in addition to longitudinal and lateral forces, slip also occurs in the contact area. Furthermore, he suggested that due to the asymmetry of the lateral tangential stresses caused by the division of the contact area into the slip and adhesion regions, their resultant does not pass through the center of the ellipse considered. As a result, the moment of the lateral force around the center of the ellipse is not equal to zero. Kalker provided expressions for calculating the tangential components of the sliding forces and the scalar moment at the wheel-rail contact point concerning the normal to the common contact plane. Pseudo-slip coefficients are determined according to the elasticity theory, depending on the ratio of the semi-axes a/b of the wheel-rail contact ellipse and the Poisson ratio. It should be noted that slip is not considered in other theories, such as Johnson and Vermeulen's theory. Moreover, the existence of actual slip and adhesion areas on the contact surface of the wheel and rail has not yet been experimentally proven. It is, essentially, a mathematical abstraction.

The presented research in this article includes comparative results that highlight the significance of the proposed solution. The ratio of the semi-axes a/b of the contact ellipse in the flange-rail contact can be greater than in the main wheel-rail contact. Nevertheless, the slip coefficients determined using Kalker's theory for slip calculation are significantly smaller than those of longitudinal and lateral slips. Therefore, the authors consider that it is possible to neglect slips in the calculations.

To evaluate the improvement achieved by using PKS wheels, it is necessary to determine the components of mechanical energy losses during the motion of the vehicle, in terms of mechanical efficiency. As is well known, the overall resistance to the motion of a railway vehicle consists of primary and additional motion resistance. According to literary sources, the primary resistance components to the motion of a railway vehicle include bearing friction, rolling friction of the wheels on the rails, sliding friction of the wheels on the rails, aerodynamic drag, energy dissipation resistance into the track, and energy dissipation resistance into the surrounding environment. The calculation of these motion resistance components is a relatively demanding task. Moreover, the values of some components, such as energy dissipation into the track and the surrounding environment, depend on a large number of factors and can only be accurately determined experimentally.

## Conclusions

The reduction of energy loss and improvement of energy efficiency remains significant issue in the railway industry. One effective approach to achieving this is to minimize kinematic running resistance. In this study, we conducted simulation computations of an urban rail vehicle running with two different types of wheels: the TKS and PKS wheels. The main indicators affecting the magnitude of creep forces in the wheel/rail contacts were calculated through these simulations. Specifically, we computed the magnitudes of the components of the creep forces and sliding speed in the wheel/rail contact points. Our results indicated that the use of PKS wheels in urban rail vehicle undercarriages resulted in a redistribution of creep force components acting in the wheel/rail contact points, compared to TKS wheels. This, in turn, affected the amount of mechanical energy dissipated in these contacts.

A comparative analysis of the simulation modelling results for an urban rail vehicle running on a chosen railway track indicates that the utilization of PKS wheels in the undercarriages of urban rail vehicles can reduce the level of kinematic running resistance and minimize the loss of mechanical energy dissipated in the wheel/rail contacts by up to 40%. Such a significant reduction in running resistance could result in more efficient operation of trams in the future.

## Supplementary Information

Supplementary Information.

## Data Availability

All data generated or analysed during this study are included in this published article.

## List of symbols

oCV: the central vertical axis of an undercarriage
IRWs: independently Rotating Wheels
RW: a rigid axle
PKS: the Perspective Construction Scheme
TKS: the Traditional Constructive Scheme
T_x_: a longitudinal component of the creep force [N]
T_y_: a lateral component of the creep force [N]
v_x_: a longitudinal component of the slip velocity [m/s^2^]
v_y_: a lateral component of the slip velocity [m/s2]
v: running speed [m/s^2^]
R: a curve radius [m]
E_abi_: mechanical energy [J]
A_abi_: work of the creep forces [J]
a: a number of a wheelset in the running direction
b: a side of an urban rail vehicle
$T_{{abi}}^{x}$: a lateral component of the creep forces in the i-th wheel/rail contact [N]
$T_{{abi}}^{y}$: a lateral component of the creep forces in the i-th wheel/rail contact [N]
$S_{{abi}}^{x}$: the mean slippage in the i-th wheel/rail contact of the corresponding wheel in the longitudinal direction
$S_{{abi}}^{y}$: the mean slippage in the i-th wheel/rail contact of the corresponding wheel in the lateral direction
$v_{{x_{{abi}} }}$: a component of the sliding speed in each contact in the longitudinal direction [m/s^2^]
$v_{{y_{{abi}} }}$: a component of the sliding speed in each contact in the lateral direction [m/s^2^]
t: the simulation period [s]
f: the sampling frequency [Hz]
$A_{\Sigma }$: the total expenditure of mechanical energy during sliding in the wheel/rail contact [J]
$\overline{N}$: the average power dissipated during the wheels sliding on the rails [W]
