# Supplementary material for: Kinematic running resistance of an urban rail vehicle undercarriage: a study of the impact of wheel design
Source: Sci Rep. 2023 Jul 5;13:10856. doi: 10.1038/s41598-023-37640-w (PMC10322867; doi:10.1038/s41598-023-37640-w)
Supplement: Supplementary file 1 — Supplementary Information. [file 41598_2023_37640_MOESM1_ESM.docx]

**The Research of Kinematic Running Resistance of an Urban Rail Vehicle Undercarriage Depending on the Railway Wheels’ Designs**

Stanislav Semenov^1^, Evgeny Mikhailov^1^, Maxim Kovtanets^1^, Oksana Sergienko^1^, Ján Dižo^2^*,
Miroslav Blatnický^2^, Juraj Gerlici^2^, Mariusz Kostrzewski^3^

^1^Volodymyr Dahl East Ukrainian National University, Faculty of Transport and Building, Department of Logistics and Traﬃc Safety, Ioanna Pavla II str., 17, 01042 Kyiv, Ukraine

^2^Department of Transport and Handling Machines, Faculty of Mechanical Engineering, University of Žilina, Univerzitná 8215/1, 010 26 Žilina, Slovak Republic

^3^Division for Construction and Operation of Transport Means, Faculty of Transport, Warsaw University of Technology, Koszykowa 75, 00-662 Warsaw, Poland

*Corresponding author

Stanislav Semenov: [semenov@snu.edu.ua](mailto:semenov@snu.edu.ua), ORCID: <https://orcid.org/0000-0002-5236-4557>

Evgeni Mikhailov: [mihajlov@snu.edu.ua](mailto:mihajlov@snu.edu.ua), ORCID: <https://orcid.org/0000-0002-6667-5348>

Maxim Kovtanets: [kovtanec@snu.edu.ua](mailto:kovtanec@snu.edu.ua), ORCID: <https://orcid.org/0000-0002-6804-7214>

Oksana Sergienko: [sergienkooksana@snu.edu.ua](mailto:sergienkooksana@snu.edu.ua)

Ján Dižo: [jan.dizo@fstroj.uniza.sk](mailto:jan.dizo@fstroj.uniza.sk), ORCID: <https://orcid.org/0000-0001-9433-392X>

Miroslav Blatnický: [miroslav.blatnicky@fstroj.uniza.sk](mailto:miroslav.blatnicky@fstroj.uniza.sk), ORCID: <https://orcid.org/0000-0003-3936-7507>

Juraj Gerlici: [juraj.gerlici@fstroj.uniza.sk](mailto:juraj.gerlici@fstroj.uniza.sk), ORCID: <https://orcid.org/0000-0003-3928-0567>

Mariusz Kostrzewski: [mariusz.kostrzewski@pw.edu.pl](mailto:mariusz.kostrzewski@pw.edu.pl), ORCID: <https://orcid.org/0000-0001-5078-4067>

**Conflicts of Interest:**

The authors declare no conflict of interest.

**Ethical approval:**

This work was prepared according to the rules of good practice.

**Ethical statement:**

This work was carried out in accordance with Publishing Ethics.

**Data availability:**

All data generated or analysed during this study are included in this published article.

**Funding:**

“This publication has been supported by the project KEGA 031ŽU-4/2023: Development of key competencies of the graduate of the study program Vehicles and Engines.”

“This publication was realized with support of Operational Program Integrated Infrastructure 2014–2020 of the project: Innovative Solutions for Propulsion, Power and Safety Components of Transport Vehicles, code ITMS 313011V334, co-financed by the European Regional Development Fund.”
